# Serum fetuin A and cystatin B as biomarkers among autism spectrum disorder patients in central India

**DOI:** 10.6026/973206300220733

**Published:** 2026-02-28

**Authors:** Sameeksha Luthra, Niranjan Gopal, Urmila Dahake, Dnyanesh Amle, Jyoti E John, Roshan Takhelmayum, Utsav Haldar, Jancy Jose, Chandrasekaran Kirubhanand

**Affiliations:** 1 Department of Biochemistry, All India Institute of Medical Sciences, Nagpur, Maharashtra, India; 2 Department of Paediatrics, All India Institute of Medical Sciences, Nagpur, Maharashtra, India; 3 Department of Anatomy, All India Institute of Medical Sciences, Nagpur, Maharashtra, India

**Keywords:** Autism spectrum disorder (ASD), Cystatin B, Fetuin-A, Biomarkers, Neuroinflammation, India, Paediatric Neurodevelopment, Enzyme-Linked Immunosorbent Assay (ELISA), ROC Analysis

## Abstract

Autism Spectrum Disorder (ASD) remains underdiagnosed in low- and middle-income countries due to reliance on subjective behavioural assessments and lack of validated objective biomarkers for early detection. Therefore, it is of interest to investigate the potential of two serum biomarkers, Fetuin-A and Cystatin B, as objective diagnostic indicators for ASD in 60 children from Central India (30 with ASD, 30 controls). While Fetuin-A showed a non-significant trend of elevation in the ASD group, Cystatin B levels were found to be significantly elevated in the ASD subjects compared to the neurotypical controls. ROC analysis demonstrated moderate diagnostic potential for both, with Cystatin B showing a higher Area under the Curve (AUC) of 69.5%. Thus, Cystatin B is a promising serum biomarker that could supplement current diagnostic tools for the early and accurate identification of ASD.

## Background:

Autism Spectrum Disorder (ASD) represents a diverse group of neurodevelopmental conditions that typically emerge in early childhood and are marked by persistent challenges in social communication, along with restricted and repetitive patterns of behaviour [1]. The disability-adjusted life years (DALYs) attributed to ASD reached 4.3 million that same year, indicating not only high prevalence but also substantial long-term functional impairment [2]. A consistent male-to-female ratio of approximately 4:1 is observed worldwide and in India, with the INCLEN study (2019) estimating the prevalence of ASD at approximately 1.12% among children aged two to nine years, equivalent to one in every 68 children, reflecting both improved awareness and a genuine rise in incidence [3]. However, a significant proportion of ASD cases in India remain undiagnosed or misdiagnosed, particularly those with milder symptoms and furthermore the reliance on hospital-based data further skews prevalence estimates [4, 5]. Diagnostic challenges are compounded by limited use of validated, culturally adapted tools, poor healthcare access and social stigma-factors that delay timely identification and intervention [2]. The diagnosis of ASD is based on standardized behavioural criteria defined by the DSM-5 and implemented globally through tools such as the Autism Diagnostic Observation Schedule (ADOS) and the Autism Diagnostic Interview-Revised (ADI-R) [6]. In India, clinical assessment is conducted using nationally validated tools like the Indian Scale for Assessment of Autism (ISAA) and the INCLEN Diagnostic Tool for ASD (INDT-ASD), which are widely used in both clinical and certification settings [6, 7]. Although these tools are effective, they are fundamentally observational and depend heavily on clinical expertise and caregiver reporting introducing subjectivity, often delaying or missing diagnosis in children with mild or atypical presentations which underscores the need for objective, accessible biomarkers that can supplement current methods and enable earlier, more accurate detection of ASD [7]. To address this diagnostic gap, our study focuses on evaluating Fetuin-A and Cystatin B as potential serum biomarkers, based on their involvement in neurodevelopment and immune-inflammatory pathways known to be altered in ASD. Fetuin-A (Fet-A), also known as α2-Heremans-Schmid glycoprotein (AHSG), is a 52 kDa plasma glycoprotein predominantly synthesized in the liver which functions as a multifunctional regulatory protein involved in calcium metabolism, insulin signaling, adipogenesis and inflammatory modulation [8]. Its circulating levels have been associated with metabolic disorders, cardiovascular disease and inflammatory conditions [9, 10- 11]. Emerging evidence also points to its possible involvement in neuroinflammatory pathways relevant to ASD, indicating that altered Fet-A levels may reflect underlying biochemical changes in affected individuals [12, 13]. Cystatin B (CSTB) is a small, 11 kDa cytosolic proteins belonging to the family-1 cysteine protease inhibitors, characterized by its monomeric, single-chain structure and widespread expression across tissues [14]. CSTB plays important roles in neuronal protection, synaptic plasticity and regulation of inflammatory responses-functions that are critically relevant to neurodevelopment [15, 16-17]. Recent evidence suggests its involvement in central nervous system physiology, with CSTB localized to synaptic regions and implicated in brain plasticity and immune modulation [16]. Although its exact role in ASD remains to be elucidated, emerging findings indicate a possible association between CSTB expression and autistic traits, supporting its potential utility as a neurodevelopmental biomarker [18]. Therefore, it is of interest to serum Fetuin-A and Cystatin B as biomarkers for autism spectrum disorder in Central India.

## Materials and Methods:

## Participants:

This cross-sectional study was carried out over a period of eight months, from June 2024 to January 2025. A total of 60 children aged 3 to 12 years were enrolled through the paediatric outpatient department, comprising 30 children clinically diagnosed with ASD and 30 age- and sex-matched typically developing controls. The diagnosis of ASD was established using the Indian Scale for Assessment of Autism (ISAA), administered by trained professionals and supplemented by relevant clinical history. Additional information on social and demographic variables was obtained from caregivers through a structured data collection tool. Participants with ASD associated with obsessive-compulsive disorder, epileptic seizures, affective disorders, fragile X syndrome, cognitive development delay, or any additional psychiatric or neurological diseases were not included in the study. Similarly, subjects with any major illness conditions were not included as controls. The study was approved by the Institutional Ethics Committee. A well-written and informed consent was obtained from all participants. The Declaration of Helsinki (1964) and its 2013 amendment were followed while conducting the study.

## Sample collection and analysis:

Non-fasting venous blood samples were collected from all the subjects meeting the inclusion criteria in 3 mL plain (red top) vacutainer tubes for blood collection. The samples were centrifuged directly after the blood sampling for 10 minutes at 3000 RPM. Until analysis, the serum samples were kept at -80°C. Serum concentrations of Fetuin-A and Cystatin B were measured using commercially available enzyme-linked immunosorbent assay (ELISA) kits (Fetuin-A: E-EL-H0386: Human FETUA ELISA Kit, Elabscience; Cystatin-B: ELK1325: Human CSTB ELISA Kit, ELK Biotechnology), following the manufacturer's protocols. Standards and a few samples were run in duplicate during the analysis to ensure reproducibility. For analysis, a multimode plate reader spectrophotometer (Synergy HTX, BioTEK, USA and Gen5 3.08 software) was used. Serum Fet-A levels were calculated as μg/mL and serum CSTB levels were calculated as ng/mL.

## Statistical analyses:

The sample size was calculated using data from previous study, power of the study to be 80% and alfa error to be 5% for Fetuin A [13] and cystatin B. The sample size was calculated using PS (Power and sample size calculator vs. 3.1.6.). Data were recorded in Microsoft Excel™ (version 2022, Microsoft® Inc., USA) and all statistical analyses were performed using SPSS® for Windows™ (version 20, IBM® Corp., NY; licensed software: ANTLR 2.7.5). Demographic and clinical variables were summarized using descriptive statistics: categorical data were presented as frequencies and percentages, while continuous variables were expressed as mean ± standard deviation (SD) or median with range, depending on data distribution. To compare differences between the ASD and control groups, Student's unpaired t-test was used for age, Fetuin A and Cystatin B. The qualitative variables such as gender, socioeconomic status were compared using Chi-square test or Fisher's exact test, as appropriate. Correlation analysis between ASD severity and biochemical parameters was carried out using Pearson's correlation coefficient. ROC curve was plotted to access the clinical significance of biomarkers. A p-value of <0.05 was considered statistically significant for all tests.

## Results and Discussion:

A total of 60 paediatric participants were enrolled in the study, including 30 children with ASD and 30 age- and sex-matched neurotypical controls. Table 1 depicts the age distribution across cases and controls. All the recruited participants fall within the 3-9 years age range. Among ASD cases, the highest proportion of subjects belonged to the 3-4 years age group (n = 18, 60%), followed by the 5-6 years age group (n = 8, 26.67%). In contrast, within the control group, the greatest number of participants were five years of age (n = 7, 23.33%), followed by those aged 3 years (n = 6, 20%). However, the mean age of participants in the ASD group was 4.56 ± 1.54 years, while in the control group was 5.30 ± 1.72 years. The analysis indicated no significant difference between the groups (p = 0.088), confirming the homogeneity of age distribution. Similarly, the study population demonstrated an equal gender distribution (p=1.0) across both groups, with 24 males (80%) and six females (20%) in each, showing male predominance. The socioeconomic status of the study participants was assessed, with the highest proportion in both the ASD and control groups belonging to the high socioeconomic category. Additionally, the study groups were also matched for their dietary habits (p=0.852). Table 1 shows the demographic characteristics of study population. *: Student's unpaired t-test was used for analysis. **: Chi square test was used for analysis.

**Figure 1:**
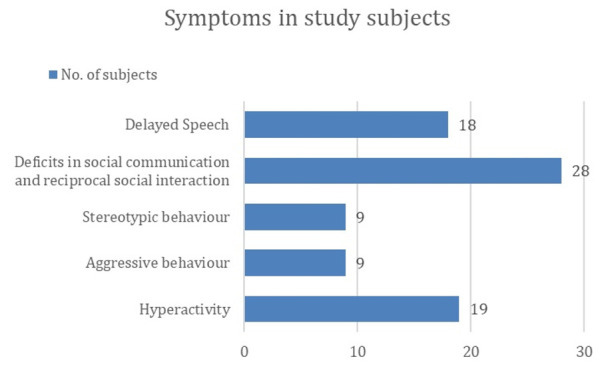
Symptom profile of cases

**Figure 2:**
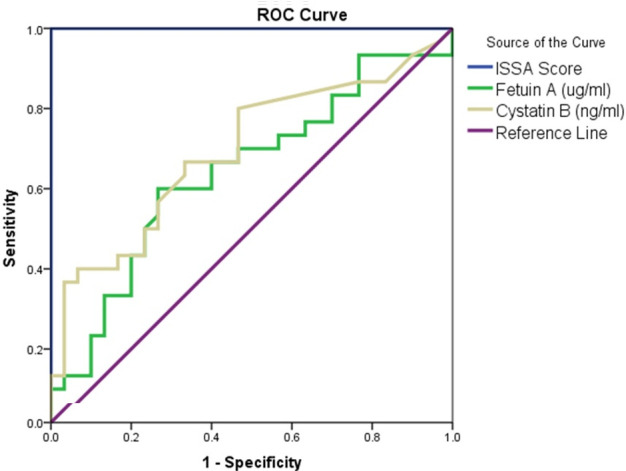
Comparative ROC curve analysis of Fetuin-A and Cystatin-B

**Figure 3:**
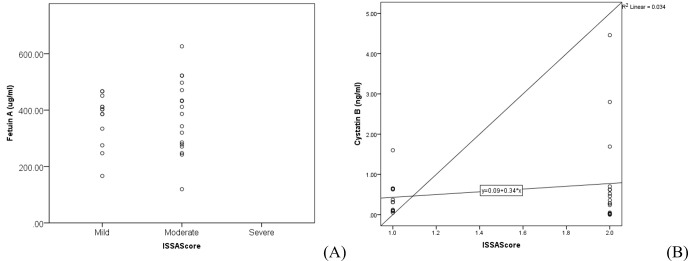
Scatter plot of Fetuin-A (a), Cystatin-B (b) and ISSA score

Figure 1 present the distribution of clinical symptoms observed in ASD cases. The most frequently reported symptom was deficits in social communication and reciprocal social interaction, observed in 28 (93.3%) of the cases, followed by hyperactivity, which was noted in 19 (63.3%) of the subjects. Delayed speech was present in 18 (60%), while aggressive behaviour and stereotypic behaviour were identified in nine (30%) of the cases. The mean Fetuin-A level in ASD cases was 373.91 ± 112.73 µg/mL, whereas in controls was 321.43±96.35 µg/mL. However, no significant difference was noted between the study groups regarding Fetuin-A levels (p=0.059). Whereas, the median Cystatin-B level in ASD cases was 0.335 ng/mL (range: 0.0-4.46 ng/mL), compared to 0.43 ng/mL (range: 0.0-0.81 ng/mL) in controls. The Cystatin-B was found to be significantly elevated in subjects with ASD compared to control subjects (p=0.009). ROC curve was plotted to assess the diagnostic significance of Fet-A and Cystatin-B Figure 2. AUC was found to be 64.2% for Fetuin-A and 69.5% for Cystatin-B. The sensitivity of Fetuin-A was found to be 70% and specificity 43% at a cut-off value of 289.86. The sensitivity of Cystatin-B was found to be 67% and specificity 67% at a cut-off value of 0.22. Pearson correlation analysis indicated no statistically significant correlation between serum marker levels and disease severity Figure 3.

The present study implemented a cross-sectional methodology to investigate the serum concentrations of Fetuin-A and Cystatin-B in the subjects with ASD in Central India and systematically compared these values with those of the control population. While Fetuin-A levels were higher in the ASD group than in controls, the difference did not reach statistical significance. In contrast, Cystatin B levels were significantly elevated in children with ASD, demonstrating moderate sensitivity and specificity in ROC analysis. The observed male predominance in both study groups aligns with global epidemiological trends in ASD. Although the participants were matched for age and gender, most belong to higher socioeconomic strata, a factor associated with increased diagnostic access and awareness. The clinical symptom profile among ASD cases-predominantly impaired social interaction, delayed speech, hyperactivity and stereotyped behaviours- reflects the known heterogeneity of ASD manifestations. Fetuin-A is a multifunctional glycoprotein involved in inflammatory regulation, neurodevelopment and metabolic processes. Contrary to previous studies by Kurt *et al.* (2021) [12] and Al-Awadhi *et al.* (2022) [13], which reported lower serum Fetuin-A levels in children with ASD, our study identified elevated levels in affected individuals. This discrepancy may stem from differences in population, study design, or biological matrices (serum versus plasma). The elevated Fetuin-A in our cohort could suggest a compensatory anti- inflammatory response or altered hepatic regulation in ASD. However, given the borderline statistical significance (p=0.059), further validation is required in larger cohorts. Cystatin B, a protease inhibitor with roles in synaptic regulation, immune modulation and neuronal integrity, showed a statistically significant increase in ASD cases. These findings support and extend previous work by Smedler *et al.* (2021) [18], who identified CSTB as strongly associated with autistic traits. The elevated CSTB levels in our study may reflect underlying neuroimmune disturbances and cellular stress responses, mechanisms increasingly implicated in ASD pathophysiology [16]. Although no significant correlation was observed between biomarker levels and ASD severity, both Fetuin-A and Cystatin B demonstrated potential diagnostic relevance, with moderate sensitivity and specificity in ROC analysis. These findings underscore the promise of serum-based biomarkers in aiding earlier and more objective diagnosis of ASD. Nonetheless, this study has limitations, including a relatively small sample size and a cross- sectional design. Larger, multi-centric and longitudinal studies are warranted to explore the diagnostic and prognostic utility of these markers and to better understand their roles in the neurobiology of ASD.

## Conclusion:

We show that both serum fetuin A as well as Cystatin B are elevated in subjects with ASD. However, the elevation of cystatin B is statistically significant indicating that cystatin B can be used as both diagnostic and predictive marker whereas, serum fetuin A can be a better predictive marker but not as a diagnostic marker.

## Advancement to knowledge:

This study provides novel evidence from Central India that serum Cystatin B is significantly elevated in children with Autism Spectrum Disorder (ASD) and demonstrates moderate diagnostic accuracy, supporting its potential role as an objective, minimally invasive adjunct biomarker to complement behaviour-based diagnostic tools. While previous studies have implicated neuroinflammation, immune dysregulation and synaptic dysfunction in ASD pathophysiology (2020-2025), human clinical data on circulating Cystatin B in ASD remain scarce, particularly in low- and middle-income settings. Our findings extend emerging evidence linking CSTB to autistic traits and neuroimmune pathways and provide population-specific data from India, where objective biomarkers are urgently needed to reduce diagnostic delay. In contrast to earlier reports of reduced Fetuin-A in ASD from other populations, this study observed higher (though not statistically significant) Fetuin-A levels, highlighting geographical and biological heterogeneity in inflammatory-metabolic responses in ASD. This underscores the need for context-specific biomarker validation before clinical translation. Overall, this study advances knowledge by providing first clinical evidence from Central India on serum Cystatin B in ASD, supporting Cystatin B as a promising adjunct diagnostic biomarker, demonstrating population-specific variation in Fetuin-A, emphasizing the need for regionally validated biomarker panels and reinforcing the role of neuroimmune dysregulation as a measurable peripheral signature of ASD.

## Figures and Tables

**Table 1:** Demographic profile and biomarker levels in cases and controls

**Parameter**		**Control (n=30)**	**ASD (n=30)**	**t/x2**	**p value**
Age (Years)*		5.3 ± 1.725	4.56 ± 1.546	1.734	0.088
Gender**	Male	24 (80 %)	24 (80 %)	0	1
	Female	6 (20%)	6 (20%)		
Socioeconomic status**	High	25 (83.34%)	27 (90%)	2.077	0.354
	Middle	3 (10%)	3 (10%)		
	Low	2 (6.67%)	0		
Diet**	Vegetarian	11 (36.67%)	13 (43.3%)	0.321	0.852
	Eggetarian	5 (16.67%)	5 (16.67%)		
	Non-vegetarian	14 (46.67%)	12 (40.0%)		
Fetuin A (µg/mL)*		321.43 ± 96.35	373.91 ± 112.73	577.5	0.059
Cystatin B (ng/ml)*		0.05 (0.0-8.1)	0.335 (0.0-4.46)	625.5	0.009
